# A Lipopolysaccharide from *Pantoea Agglomerans* Is a Promising Adjuvant for Sublingual Vaccines to Induce Systemic and Mucosal Immune Responses in Mice via TLR4 Pathway

**DOI:** 10.1371/journal.pone.0126849

**Published:** 2015-05-15

**Authors:** Masahiro Fukasaka, Daisuke Asari, Eiji Kiyotoh, Arimichi Okazaki, Yasuyuki Gomi, Takeshi Tanimoto, Osamu Takeuchi, Shizuo Akira, Mitsuhiko Hori

**Affiliations:** 1 Life Science Research Center, Corporate Research & Development Division, Nitto Denko Corporation, Ibaraki, Osaka, Japan; 2 Research and Production Technology Department, The Research Foundation for Microbial Diseases of Osaka University, Kanonji, Kagawa, Japan; 3 Laboratory of Infection and Prevention, Institute for Virus Research, Kyoto University, Shogoin Kawara-cho, Sakyo-ku, Kyoto, Japan; 4 Laboratory of Host Defense, WPI Immunology Frontier Research Center (IFReC), Research Institute for Microbial Diseases, Osaka University, Suita, Osaka, Japan; Imperial College London, UNITED KINGDOM

## Abstract

A lipopolysaccharide from *Pantoea agglomerans* (LPSpa) has been applied to various fields for human use as a Toll-like receptor 4 ligand and its safety has been confirmed. Here, we showed for the first time the application of LPSpa as an effective mucosal adjuvant for activating vaccine-induced antigen specific immune responses. Mice sublingually immunized with influenza vaccine (HA split vaccine) with LPSpa induced both HA-specific IgG (systemic) and IgA (mucosal) antibody responses, which led to a significant increase in survival rate against lethal influenza virus challenge compared with subcutaneous vaccination. After sublingual administration of ovalbumin with LPSpa, ovalbumin-specific mucosal IgA responses were induced at both mucosal surfaces close to the immunized site and at remote mucosal surfaces. Sublingual administration of LPSpa evoked local antigen-uptake by dendritic cells in cervical lymph nodes. LPSpa induced cytokine production and the maturation and proliferation of innate immune cells via Toll-like receptor 4 in dendritic cells. Collectively, these results suggest that LPSpa can be used as an effective mucosal adjuvant to stimulate and activate local innate immune cells to improve and enhance mucosal vaccine potency against various pathogens.

## Introduction

Almost all environmental pathogens enter the body through mucosal surfaces including the respiratory, gastrointestinal, and genital tracts that act as the first line of defense. Therefore, mucosal vaccination is one of the most effective prophylaxes to prevent infectious diseases because it elicits both mucosal and systemic immune responses [1–3]. The sublingual (s.l.) administration of antigens has been well documented for allergen desensitization therapy [4, 5]. The s.l. route has recently been demonstrated to be an attractive site for vaccination against various bacterial and viral diseases [6–10] because antigens are not exposed to degradation caused by gastrointestinal tract [11] and are prevented from being redirected to the central nervous system [12–14].

For inactivated antigens, sufficient immune responses that protect from infectious pathogens, especially at mucosal surfaces, can be enhanced by co-administration with adjuvants (immunostimulation). S.l. administration of inactivated influenza vaccine (A/PR/8) with a subunit of mutant cholera toxin (CT) and B subunit of a heat-labile toxin (LT) (mCTA/LT) induced humoral immune responses and protected against influenza virus infection [13], these enterotoxins, CT and LT, are able to act as adjuvants [15–17]. Local IgA responses and salivary hemagglutination inhibition (HI) responses were elicited by s.l. vaccination of influenza H5N1 virosomes in combination with a bacterial second messenger, c-di-GMP [18]. Moreover, some Toll-like receptor (TLR) ligands are potential mucosal adjuvants for HIV gp140 and tetanus toxoid [19]. These findings suggest that vaccination by the s.l. route with a suitable adjuvant is an attractive method for the administration of vaccines. However, because of safety concerns, few adjuvants such as alum and monophosphoryl lipid A (MPL), have been used in humans. To develop safer adjuvants, especially for s.l. vaccination, we have focused on substances already approved for human use.

LPSpa is a lipopolysaccharide (LPS) (TLR4 ligand) derived from *Pantoea agglomerans*, a Gram-negative bacterium that grows symbiotically with wheat [20, 21]. The oral or intradermal administration of this substance, a water extract of flour or purified from *Pantoea agglomerans*, evokes innate immune cells like macrophages to prevent and treat various health disorders including pathogenic infection, type I allergic reactions and cancer [22–26]. Moreover, safety trials of the fermented flour extracts containing LPSpa as an active ingredient using reverse mutation or chromosome aberration tests, single-dose toxicity, repeated dose toxicity and other skin safety studies were performed [27]. These studies confirmed that oral and transdermal administration of LPSpa derived from fermented flour extract showed no significant toxicity at doses that evoked immune responses. Based on these features, several products were launched; for example, an application in health food to prevent and improve metabolic syndromes, skincare products to maintain the healthy state of skin and foodstuffs for stockbreeding and aquatic culture to activate innate immunity.

In this study, we evaluated the mucosal adjuvant activities of s.l. administered LPSpa in the induction of antigen-specific humoral immune responses. S.l. administration of antigens with LPSpa efficiently induced both systemic IgG and mucosal IgA production by activating innate immune cells: maturation of dendritic cells (DCs), the release of pro-inflammatory cytokines and antigen-migration. Our findings suggest that LPSpa is a potential adjuvant for the enhancement of s.l. vaccines.

## Materials and Methods

### Vaccine, adjuvant and reagents

Influenza HA split vaccines derived from A/California/07/2009 (H1N1) pdm09, A/Victoria/361/2011 (H3N2), B/Wisconsin/1/2010 and A/Puerto Rico/8/34 (H1N1) (PR8) were kindly provided from The Research Foundation for Microbial Diseases of Osaka University, Japan. PR8 viruses were prepared as previously described [28]. Ovalbumin (OVA) was purchased from Sigma Aldrich, USA. MPL was purchased from InvivoGen, USA. LPSpa was purchased from Macrophi Inc., Japan.

### Mice

Female C57BL/6 and BALB/c mice (aged 6 to 8 weeks) were purchased from SLC, Japan. The mice were reared at 23°C with a 12 h light/dark cycle and were allowed free access to standard rodent chow and water. The mice were housed for at least 5 days to adapt to their environment before experiments. All animal experiments were performed in accordance with the guidelines of the Institutional Animal Care and Use Committee of Research Institute for Microbial Diseases, Immunology Frontier Research Center of Osaka University and Nitto Denko Corporation, who specifically approved this study (Permit Number: C13H, C13I, C14E and C14F). Especially, survival studies were reviewed and approved by the animal ethics committee of Immunology Frontier Research Center of Osaka University. All efforts were made to minimize suffering under the guideline adherence.

### Immunization and sample collection

Mice were immunized twice at weekly intervals under anesthesia with thiamylal and xylazine. For s.l. vaccination, forceps were placed under the tongue, the mouth was opened wide and 30 μl of vaccine solution was administered by micropipette. Seven days after the last immunization, serum and mucosal secretions (nasal washes, bronchial lavage fluids [BALF], vaginal washes and fecal extracts) were collected. Nasal, bronchial lavage and vaginal mucosa were washed with 200, 500 or 150 μl of phosphate buffered saline (PBS), respectively. Fecal pellets were suspended in PBS (100 mg/ml) and extracted by vortexing for 10 min. After centrifugation at 3000 ×*g* for 10 min the supernatants were used as fecal extracts. For a longitudinal study to assess the persistence of antigen-specific immune responses, at 1, 4, 8 and 12 weeks after the last immunization, serum and nasal washes were collected.

### Antigen-specific IgG and IgA enzyme-linked immunosorbent assay

The titers of antigen-specific antibodies in serum and mucosal samples were determined. Immunoplates were coated with influenza vaccine (H1N1, H3N2 or B at 2.5 μg/ml) or OVA at 100 μg/ml in advance. After blocking with BlockAce (Dainippon Sumitomo Pharmaceutical, Japan), two-fold serial dilutions of the samples were added to the immunoplate followed by the addition of horseradish peroxidase-conjugated anti-mouse IgG or IgA (Bethyl Laboratories, USA). After adding TMB peroxide substrate (Nacalai Tesque, Japan) and stop solution, end-point titers were determined by the maximum dilution that exceeded 0.1 at an absorbance of 450 nm.

### HI assay

Serum samples were treated with RDE II (Denka Seiken, Japan) at a final dilution of 1:10. Two-fold serial dilutions of the samples were incubated with an equal volume containing 8 HA of split vaccine (H1N1). Then, chicken red blood cells (0.5% [v/v]) were added and incubated. HI titers were determined by the maximum dilution that showed non-agglutination.

### Virus infection

Seven days after the last immunization, mice were intranasally infected with 15 μl of PR8 virus (8.2×10^−1^ HA unit: 100×LD_50_) under anesthesia with thiamylal and xylazine. Survival rate of experimental mice is monitored daily, and their body weights were also measured every two days. Mice with a weight loss of more than 30% of the starting body weight were considered as dead and were humanely euthanized by cervical dislocation. At 5 days after virus challenge, lungs of some mice were harvested and fixed with 4% formalin. Lung sections embedded in paraffin wax were stained with hematoxylin-eosin (Wako, Japan).

### Cells and stimulation

Peritoneal macrophages: at 3 days after intraperitoneal injection of 2 ml of 4% (w/v) thioglycollate medium (Sigma Aldrich, USA), the mice were sacrificed and peritoneal exudate cells were isolated by washing with PBS. Bone-Marrow derived DCs (BMDCs): mice were sacrificed and bone marrow cells were harvested from the femurs and tibias by flushing the marrow cavity with PBS and then cultured with complete RPMI 1640 (supplemented with 10% fetal calf serum [FCS], 50 mM 2-ME, 1% penicillin [100 U/ml], 1% streptomycin [100 U/ml]) and 10 ng/ml mouse granulocyte-macrophage colony-stimulating factor (GM-CSF, PeproTech, USA) for 6 days as previously described [29]. Cells were collected and 1×10^6^ cells/ml were stimulated with LPSpa or MPL for the indicated times and concentrations. To assess the functional role of TLR2 and TLR4, peritoneal macrophages were incubated with 1 μg/ml of anti-TLR2 monoclonal antibody (mAb), anti-TLR4 mAb or isotype control Ab (all Abs were purchased from BioLegend, USA) for 30 min before stimulation.

### Cytokine enzyme-linked immunosorbent assay

Cell culture supernatants were assessed for concentrations of interleukin (IL)-6 and tumor necrosis factor (TNF)α by enzyme-linked immunosorbent assay (ELISA) according to manufacturer’s instructions (R&D Systems, USA).

### Flow cytometry

FITC-CD11c antibodies were purchased from BD Pharmingen, USA and APC-CD40, CD80 and CD86 were purchased from BioLegend. Cells were washed in flow-cytometry buffer (2% [v/v] FCS and 2 mM EDTA in PBS, pH 7.5), then incubated with each antibody for 20 min and washed twice with flow-cytometry buffer. The cells were analyzed by flow cytometry on a FACSCalibur instrument (BD Biosciences, USA), followed by data analysis using FlowJo v7.6.5 software (Tree Star).

### *In vivo* Ag migration assay

Alexa 647 OVA (50 μg) (Molecular Probes, USA) with or without LPSpa (5 μg) was administered sublingually under anesthesia with thiamylal and xylazine. After 18 h, cervical lymph nodes were harvested and single cell suspensions were stained with FITC-CD11c and assessed by flow cytometry.

### Proliferation assay

BMDCs were collected and stimulated with LPSpa (10 ng/ml) for 8 h. Cells were stained with propidium iodide (PI) buffer (0.4% sodium citrate, 0.03% Nonidet P-40, 0.05 mg/ml PI [Sigma] and 0.02 mg/ml RNase A) and assessed by flow cytometry.

### Statistics

All error bars indicate standard deviation (SD). Two-tailed student *t*-test was used to compare significant differences between two groups, whereas one-way ANOVA with Dunnett’s or Tukey’s test was used to compare differences among three or more groups. For survival curves, groups were compared with a log-rank test.

## Results

### LPSpa is a potent adjuvant and induces HA-specific humoral responses by s.l. immunization

**Fig 1:**
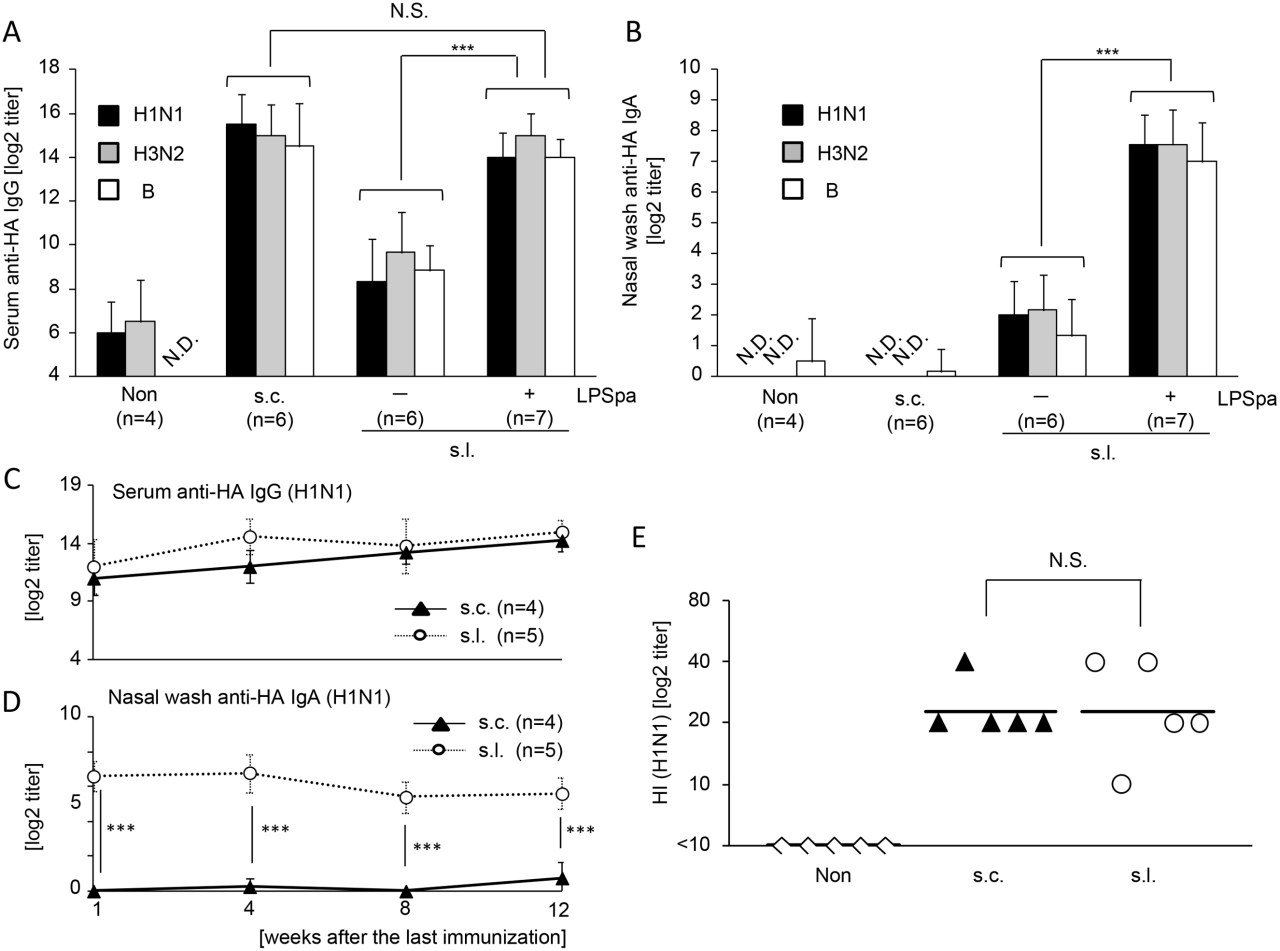
Seasonal influenza split s.l. vaccination with LPSpa induced HA-specific immune responses in serum and mucosa. BALB/c mice were divided into 4 groups and immunized twice s.c. or s.l. with H1N1, H3N2 and B strain-derived split vaccine (0.1 μg) with or without LPSpa (1 μg) at weekly intervals (n = 4–7). The titers of HA-specific (A) serum IgG and (B) nasal IgA were assessed by ELISA. In s.c. and s.l. with LPSa groups, at 1, 4, 8 and 12 weeks after the last immunization, HA (H1N1)–specific (C) serum IgG and (D) nasal IgA were assessed by ELISA (n = 4–5). (E) HI titers in 3 groups were measured in serum by HI assay (n = 5). Results were representative of at least three experiments. ***P<0.001, by one-way ANOVA with Tukey’s test (H1, H3 and B were assessed independently [A, B]). N.S., not significant. N.D., not detectable.

To clarify whether LPSpa activates immune responses as a mucosal adjuvant for s.l. vaccination against seasonal influenza viruses, we utilized HA split vaccines containing 3 strains, H1N1, H3N2 and B. These vaccine strains were selected for the 2012/13 season in Japan (IASR from National Institute of Infectious Diseases). The efficacy of such adjuvant-less split vaccines via injection was low especially for young children [30, 31]. The levels of H1N1, H3N2 and B strain specific serum IgG and mucosal IgA were determined by ELISA (Fig 1A–1D) or HI assay (Fig 1E). As shown in Fig 1A, serum HA specific IgG responses against each strain were increased in s.l. immunized mice and especially, mice immunized s.l. with vaccines containing LPSpa induced HA-specific IgG, whose levels were comparable to s.c. immunization with vaccines. The HI assay showed the same tendency as ELISA, where both s.c. and s.l. immunization with LPSpa induced sufficient production of HI Abs (H1N1) in serum (Fig 1E). In contrast, HA-specific IgA was not detected in the nasal wash after s.c. immunization compared with the non-treatment group, but mucosal vaccination induced IgA responses (Fig 1B). Moreover, s.l. immunization with LPSpa significantly enhanced HA-specific IgA responses compared with s.l. immunization without LPSpa (Fig 1B). Additionally, high levels of long lasting HA-specific Abs (H1N1) were observed until 12 weeks after the final immunization (Fig 1C and 1D). These results demonstrate that s.l. immunization with LPSpa elicited both systemic IgG comparable to s.c. immunization and mucosal IgA responses, which were not induced by s.c. immunization.

### S.l. vaccination of PR8-split vaccine with LPSpa protects mice against lethal challenge with influenza virus

**Fig 2:**
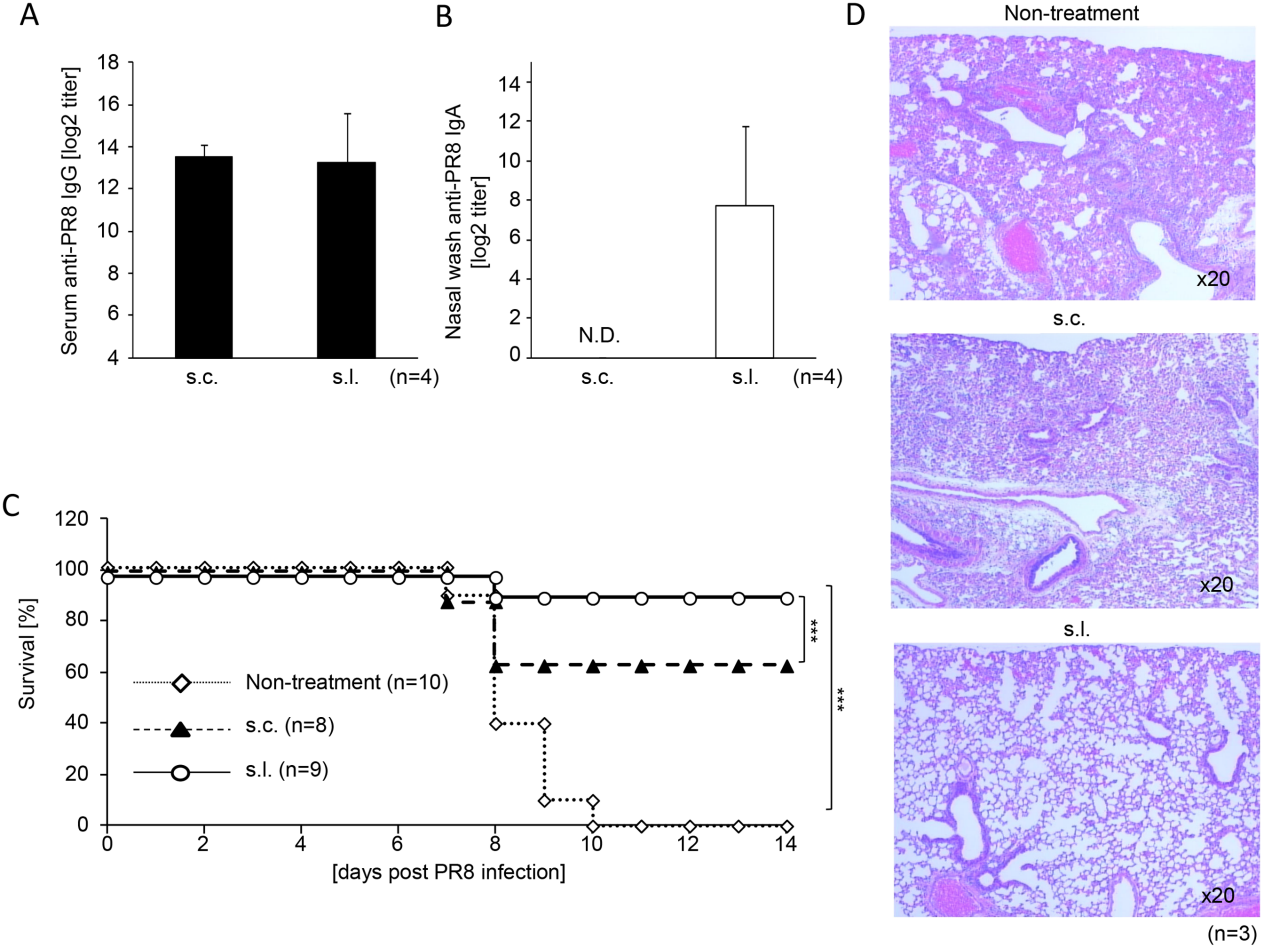
S.l. vaccination of PR8 split vaccine with LPSpa protected mice from viral challenge. BALB/c mice were divided into 3 groups and immunized twice s.c. with 0.1 μg PR8 vaccine or s.l. with PR8 vaccine (0.1 μg) and 5 μg LPSpa at weekly intervals. The titer of PR8-specific (A) serum IgG and (B) nasal IgA were assessed by ELISA (n = 4). Seven days after the last immunization, mice were challenged with a lethal dose (100×LD_50_) of PR8 virus and (C) survival rates were recorded (n = 8–10). (D) Lung sections at 5 days after virus challenge were stained with hematoxylin-eosin to evaluate cellular infiltration (n = 3). Similar experiments were performed at least three times and one representative experiment is shown. ***P<0.001, by the log-rank test. N.D., not detectable.

Next, we utilized split vaccine and viruses derived from the PR8 (H1N1) strain commonly used as a laboratory mouse-adapted influenza strain, to determine whether s.l. immunization provided protective immunity against lethal virus challenge. As seen in Fig 2A and 2B, sufficient production of PR8-specific IgG in serum was comparable between s.l. and s.c. vaccination groups but PR8-specific IgA in nasal wash was only induced efficiently by the s.l. route. Seven days after the last immunization, mice were infected intranasally with 100×LD_50_ of PR8 virus and monitored daily. As shown in Fig 2C, all non-treated mice (10/10) died within 10 days after virus infection (eight of them died by viral infection without euthanasia and 2 of them with a weight loss of more than 30% at day 8 were euthanized and recorded as dead). However, about 60% (5/8) of mice immunized s.c. survived, although transient body-weight loss was observed (data not shown). Moreover, about 90% (8/9) of mice immunized s.l. survived without any body-weight loss (data not shown). Three out of 8 and 1 out of 9 mice with vaccination groups were dead by viral infection without euthanasia. On day 5 after virus challenge, histological analysis showed that lung sections from non-treated mice and mice immunized subcutaneously exhibited severe damage caused by massive lymphocyte infiltration and inflammatory hyperemia. In contrast, lung sections from mice immunized sublingually did not induce significant lung pathology (Fig 2D). These results indicate that s.l. vaccination with LPSpa enhanced significant protective responses against lethal virus challenge due to both systemic and especially mucosal PR8 specific Ab production.

### S.l. vaccination of OVA with LPSpa induces OVA-specific immune responses at remote mucosal surfaces

**Fig 3:**
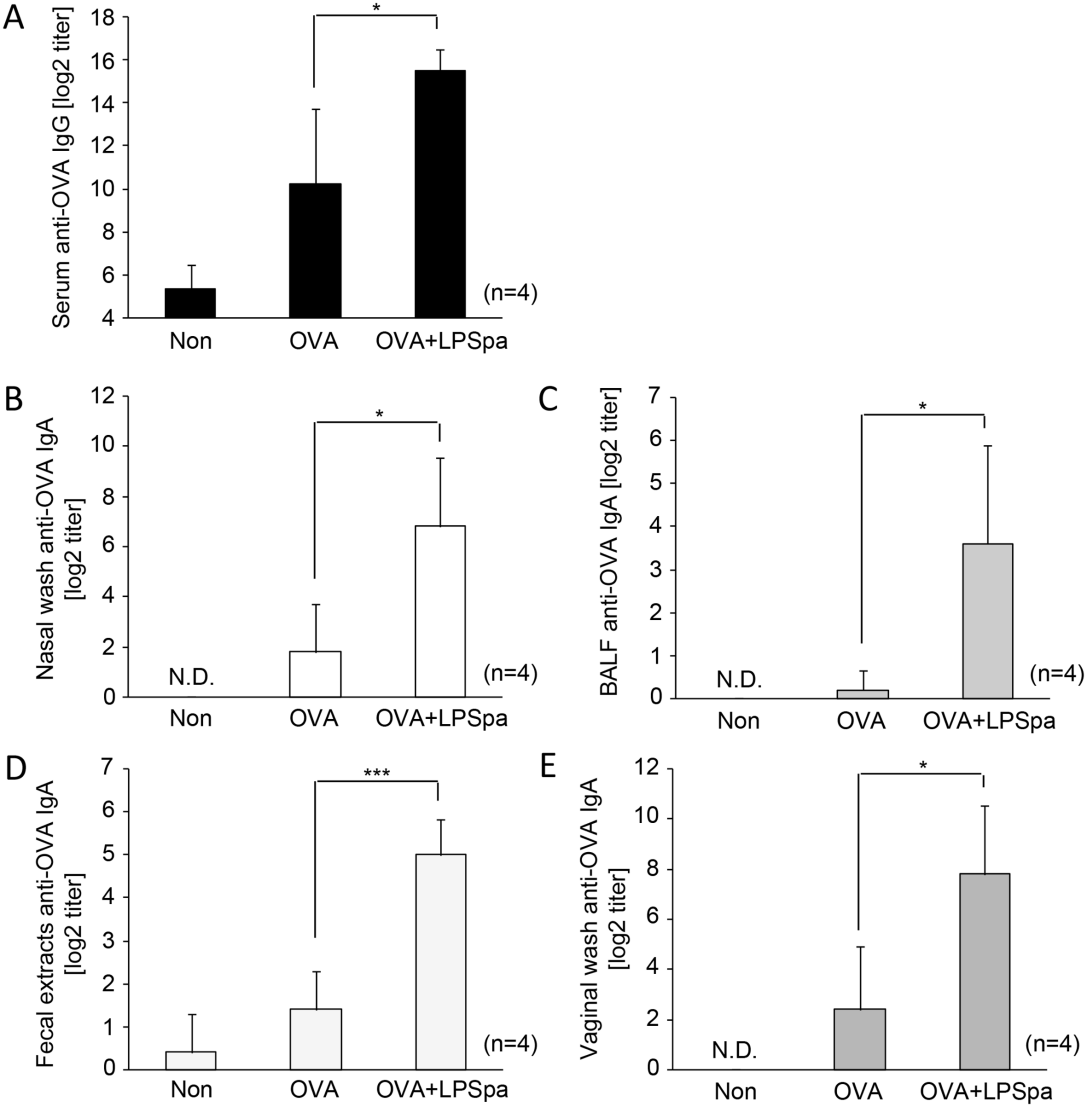
Induction of OVA-specific IgG and IgA in serum or various mucosal areas by LPSpa. BALB/c mice, divided into 3 groups, were s.l. immunized with 10 μg OVA or a mixture of OVA (10 μg) and 1 μg LPSpa twice at weekly intervals (n = 4). Seven days after the last immunization, samples were collected and the titers of (A) serum IgG, (B) nasal IgA, (C) BALF IgA, (D) fecal IgA and (E) vaginal IgA were assessed by ELISA. Results are representative of three independent experiments. *P<0.05, ***P<0.001, by one-way ANOVA with Tukey’s test. N.D., not detectable.

This study demonstrated the efficient immunization of influenza vaccine with LPSpa via oral mucosa. Because mucosal vaccination induced antigen specific IgA responses at the immunized site and at remote mucosal surfaces [32], we utilized OVA, a commonly used model antigen for vaccination, and assessed the production of OVA-specific IgA at various mucosal areas after s.l. immunization with LPSpa. As shown in Fig 3A, levels of OVA-specific IgG in serum were significantly higher when immunization contained LPSpa. Additionally, OVA-specific IgA in nasal washes as well as BALF, fecal extracts and vaginal washes were significantly induced after s.l. vaccination with LPSpa (Fig 3B–3E). These results suggest that s.l. vaccination with LPSpa elicited both systemic IgG and mucosal IgA responses in the trachea, intestine and genital tract, indicating its potential application for vaccines to defend against various pathogens such as norovirus, human immunodeficiency virus and human papilloma virus.

### S.l. administration of LPSpa activates DC migration at early stage

**Fig 4:**
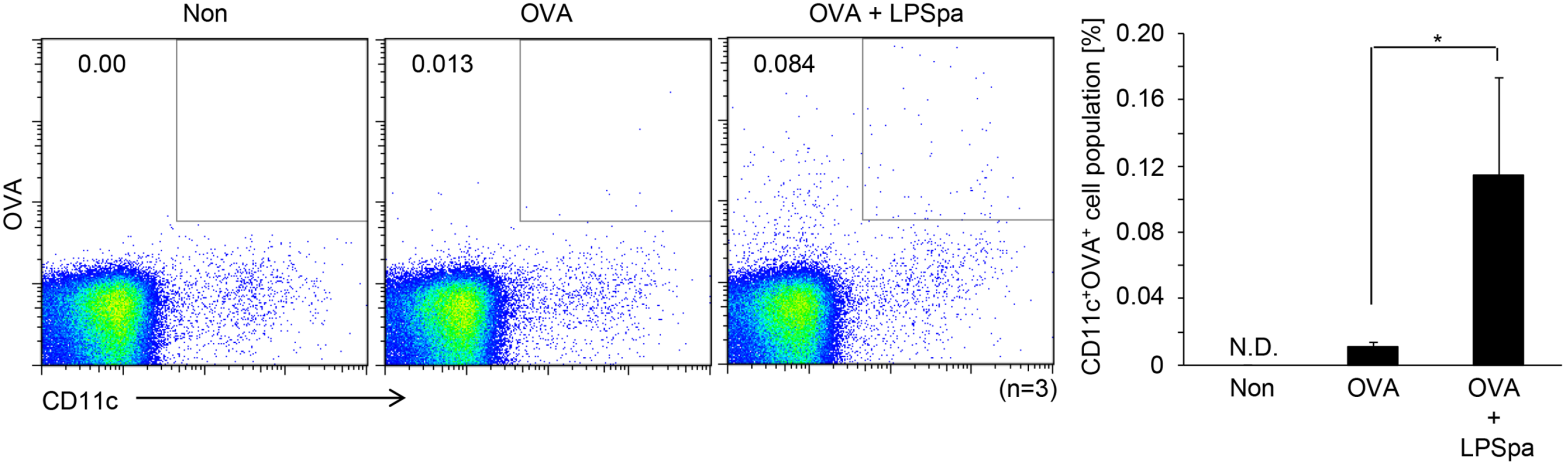
Enhanced Ag uptake of DCs in cervical lymph nodes in the presence of LPSpa. BALB/c mice, divided into 3 groups, were s.l. administered with 50 μg Alexa 647 OVA with or without LPSpa (5 μg) (n = 3). After 18 h, cervical lymph nodes were removed and cells were assessed by flow cytometry. Results are representative of three or more independent experiments. *P<0.05, by one-way ANOVA with Tukey’s test. N.D., not detectable.

After vaccination, the innate immune system is activated and DCs that function as antigen presenting cells capture antigens and migrate to adjacent local lymph nodes where they present antigens to T cells followed by the activation of the adaptive immune system [30]. To examine whether LPSpa elicited antigen uptake and migration of DCs, we administered Alexa 647-conjugated OVA (50 μg) with or without LPSpa (5 μg) and the proportion of OVA containing CD11c^+^ DCs in cervical lymph nodes was determined after 18 h. As shown in Fig 4, the number of OVA containing DCs increased in the presence of LPSpa. However, there were few OVA containing DCs in mediastinal lymph node in all groups (data not shown). These results indicate that s.l. vaccination with LPSpa can enhance the efficacy of antigen uptake and migration of DCs to cervical lymph nodes via the s.l. route leading to a link between innate and adaptive immunity.

### Innate immune cells produce cytokines, proliferate and mature after stimulation with LPSpa via TLR4

**Fig 5:**
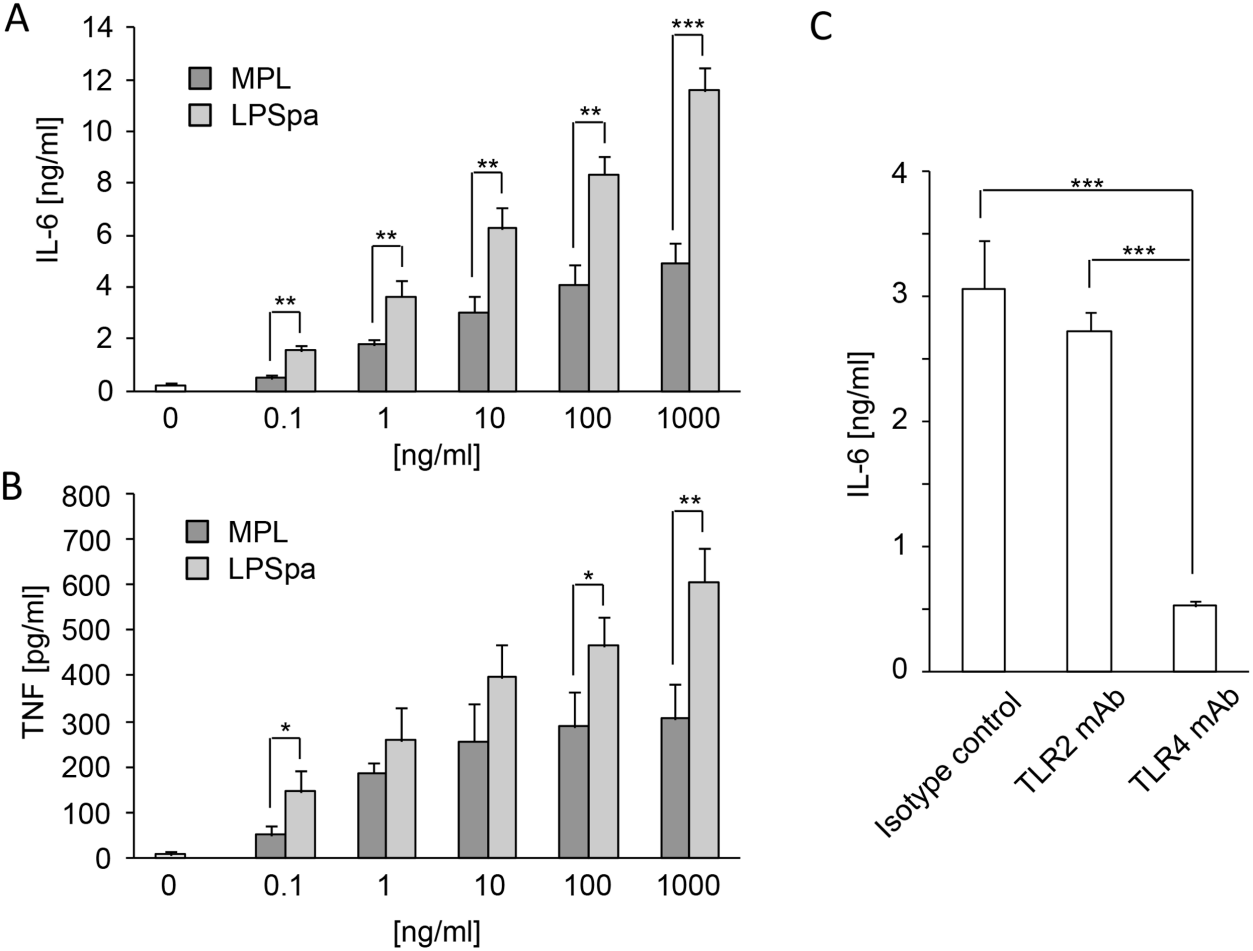
Cytokine production from mouse macrophages after stimulation with LPSpa via TLR4. Thioglycollate-elicited peritoneal macrophages were collected from BALB/c mice. Cells were stimulated for 24 h with MPL (0.1, 1, 10, 100, 1000 ng/ml) or LPSpa (0.1, 1, 10, 100, 1000 ng/ml) and (A) IL-6 or (B) TNF levels in supernatants were measured by ELISA (n = 3). Macrophages were treated with isotype Ab, TLR2 mAb or TLR4 mAb for 2 h before stimulation with LPSpa (1 ng/ml) and 24 h later, (C) IL-6 production of supernatants was measured by ELISA (n = 3). Results are representative of two (C) or three (A, B) independent experiments. *P<0.05, **P<0.01, ***P<0.001, by Two-tailed student *t*-test when comparing between two groups and one-way ANOVA with Tukey’s test when comparing between three groups.

LPSpa is a TLR4 ligand and signaling pathways triggered by TLRs lead to the production of proinflammatory cytokines, such as IL-6, IL-12p40, TNF, and type I interferons (IFNs) [33]. Additionally, signaling pathways increase the surface expression of costimulatory molecules, such as CD40, CD80 and CD86 on DCs, which are hallmarks of cellular activation [34]. Responses of peritoneal macrophages were measured after stimulation with LPSpa and MPL, another TLR4 ligand approved as an adjuvant for human vaccination, as a control. As shown in Fig 5A and 5B, the levels of IL-6 and TNF production in response to LPSpa or MPL for 24 h were increased dose-dependently, and the production of cytokines in response to LPSpa was significantly higher than for MPL at an equal concentrations. To investigate the contribution of TLR4 in cytokine production in response to LPSpa, peritoneal macrophages were treated with isotype control Ab, TLR2 mAb or TLR4 mAb followed by stimulation with LPSpa. The production of IL-6 in response to stimulation with LPSpa (1 ng/ml) was severely impaired by TLR4 mAb pretreatment (Fig 5C), indicating TLR4 signaling is critical for the production of cytokines in response to LPSpa.

**Fig 6:**
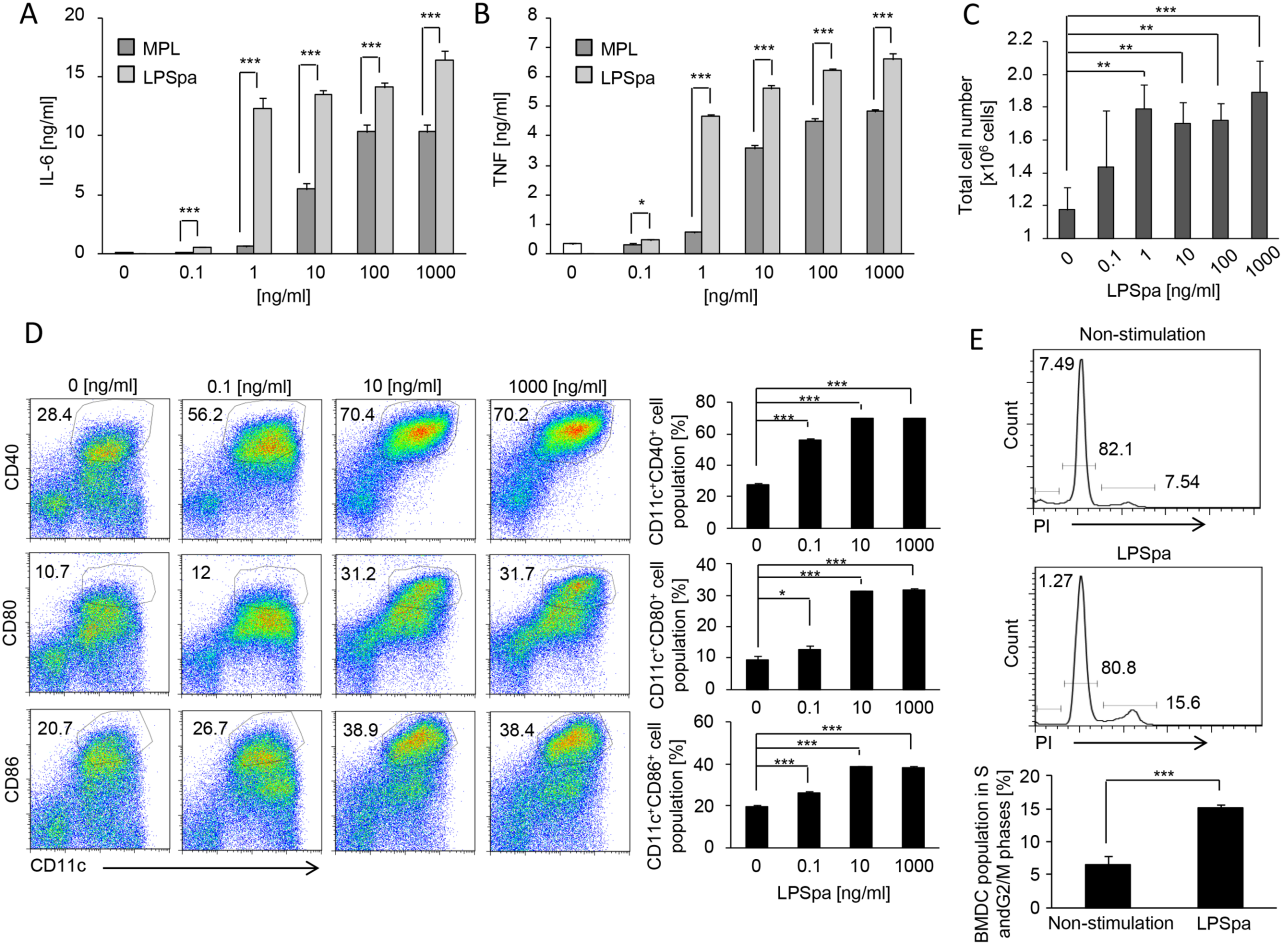
Cytokine production, proliferation and maturation of BMDCs after stimulation with LPSpa. BMDCs were obtained from C57BL/6 mice-derived bone marrow cells cultured with GM-CSF. Cells were stimulated for 24 h with MPL (0.1, 1, 10, 100, 1000 ng/ml) or LPSpa (0.1, 1, 10, 100, 1000 ng/ml) and (A) IL-6 or (B) TNF levels in supernatants were measured by ELISA (n = 3). (C) Total cell numbers were counted (n = 3) and (D) the maturation of BMDCs was assessed by measuring the expression levels of CD40, CD80 and CD86 by flow cytometry (n = 3). (E) Cells were stimulated for 8 h with LPSpa (1000 ng/ml) and then stained with propidium iodide and analyzed by flow cytometry (n = 3). Results are representative of three (C, D), four (E) or five (A, B) independent experiments. *P<0.05, **P<0.01, ***P<0.001, by Two-tailed student *t*-test when comparing between two groups and one-way ANOVA with Dunnett’s test when comparing three or more groups.

Next, we generated BMDCs by cultivating bone marrow cells in the presence of GM-CSF. As shown in Fig 6A and 6B, the production of IL-6 and TNF in response to stimulation with LPSpa was also increased similar to that in peritoneal macrophages. The number of cells expressing CD40, CD80 and CD86 was increased after stimulation with LPSpa in a dose-dependent manner (Fig 6D). Additionally, BMDCs proliferated about 1.5-fold faster than in the non-treatment group in the presence of LPSpa (0.1–1000 ng/ml) (Fig 6C). The contribution of LPSpa to cell-cycle progression in BMDCs was examined and BMDCs in S and G2/M phases were increased after stimulation with LPSpa for 8 h (Fig 6E). Taken together, these findings demonstrate that LPSpa can evoke innate immune cells such as macrophages and DCs followed by the production of proinflammatory cytokines, up-regulation of costimulatory molecules and proliferation via the TLR4 signaling pathway.

## Discussion

In this study, we discovered a novel function of LPSpa as an adjuvant that induces humoral immunity, including systemic IgG and mucosal IgA responses, after s.l. vaccination, by activating innate immune cells via TLR4.

It has been shown that s.l. administration is potentially safer than intranasal administration [13, 14]. Antigens and/or adjuvants administered intranasally can be redirected to the central nervous system through the olfactory bulbs and brain. For example, a clinical trial of intranasal vaccination of antigens and LT in Switzerland showed some cases of Bell’s palsy, and CT was reported to redirect antigens into olfactory tissues in mice [14, 35]. Such safety concerns might limit the usefulness of intranasal vaccines in humans. In contrast, after s.l. administration, vaccine components such as inactivated vaccine and live virus were not transported into olfactory bulbs and brain [13]. Therefore, mucosal priming and action mechanisms would be different between the intranasal and s.l. routes because antigen-loaded antigen presenting cells were observed mainly in mediastinal lymph nodes after intranasal administration [36], and in cervical lymph nodes after s.l. administration.

TLRs, one of the best-characterized pattern recognition receptors, are transmembrane proteins that recognize microbial components on the cell surface or in endosomes followed by the activation of innate immune cells such as macrophages and DCs [37]. On the basis of these features, many TLR ligands have been used as vaccine adjuvants. Intranasal administration of influenza vaccine with Poly(I:C) (TLR3 ligand) and/or zymosan (TLR2 ligand) induce antigen-specific IgG and IgA responses [38]. CpG ODN (TLR9 ligand) is a potent adjuvant for influenza vaccine by s.c. administration [39]. Transdermal administration of imiquimod (TLR7 ligand) enhanced both humoral and cellular immunity [40, 41]. Thus, various TLR ligands have been investigated for potential use as adjuvants, and whose effectiveness likely depends on the tissue distribution of TLRs. TLR4 is highly expressed in oral mucosal regions (vestibular/buccal region and sublingual region) and soluble forms of TLR4 and CD14 are present in human saliva and interact with oral commensal and pathogenic bacteria [42, 43]. These reports suggest that s.l. vaccination with TLR4 ligand might be an ideal vaccination strategy.

We found that LPSpa induced greater amounts of cytokines than MPL after stimulation of macrophages and DCs. MPL from *Salmonella minnesota* was used in this study as a control for TLR4 ligand. MPL has already been approved as an adjuvant in combination with AS04 (alum), in Cervarix and Fendrix produced by GlaxoSmithKline Biologicals for human papilloma virus and hepatitis B virus, respectively. However, in a previous study, s.l. immunization of HIV-1 CN54gp140 or tetanus toxoid with MPL did not induce strong antigen specific responses (IgG and IgA production) [19] probably because activation of innate immune cells by MPL was not sufficient to evoke humoral immunity.

In this study, we demonstrated that antigen-specific IgA production was strongly induced in the respiratory tract, intestine and vaginal tract after s.l. immunization with OVA and LPSpa. Previous studies have found that s.l. vaccination induced the migration of antigen-specific IgA Ab-secreting cells into the vaginal mucosa in a CCL28-dependent manner and both migratory CD8α^-^ and resident CD8α^+^ DCs were responsible for efficient priming of antigen-specific T and B cells in the cervical lymph nodes, regulated by CCL7-CCL19/CCL21 [8, 44]. It has been demonstrated that antigen-loaded DCs from s.l. mucosa recirculate to distant lymphoid organs, such as genital nodes, to activate antigen-specific CD8^+^ T cells [45]. These findings imply that LPSpa-based s.l. vaccines can be applied to protect against pathogen infection via various mucosal routes.

For the prevention of influenza virus infection, there are three different types of vaccines: live-attenuated virus vaccine, formalin-inactivated whole-virus vaccine and HA split vaccine [31]. It has been shown that HA split vaccine, currently used for seasonal vaccination in Japan to avoid adverse effects, contains only purified components (lacks virus-derived RNA [TLR7 ligand]) and does not induce innate immune responses due to the absence of TLR7/MyD88 pathway signaling [30, 31]. Therefore, after vaccination with the split vaccine, naïve mice that had never experienced influenza virus infection could not sufficiently protect against virus challenge [31]. This suggests that adjuvant activity via TLR ligands is essential for the efficient induction of adaptive immunity. Additionally, secreted IgA in the respiratory tract protected against virus infection and is important for cross-protection against heterologous influenza virus strains [46]. In this study, we demonstrated that s.l. vaccination with split vaccine and LPSpa induced efficient production of serum IgG and mucosal IgA at a relatively low vaccine dose (0.1 μg). Thus, s.l. vaccination with LPSpa against influenza virus might be a useful vaccination method.

In the survival study (Fig 2C), we determined 30% of body weight loss as a humane endpoint because 15%-35% of body weight loss and/or moribund conditions have been commonly used as humane endpoints [47–51]. However, judging from the results, it may not have been appropriate in terms of animal welfare because most of mice were dead by viral infection without euthanasia. For future work, in an influenza (especially PR8 virus) challenge study, we strongly recommend that mice which have weight loss of more than 25% of the body weight and/or are in moribund conditions such as no eating, no drinking, marked immobility and dull fur are considered to reach an experimental and a humane endpoint.

In summary, this study clearly demonstrated that LPSpa is an effective adjuvant for mucosal vaccine that can elicit humoral immunity, both systemic and local immunoresponses, after s.l. vaccination. Now we have been developing and improving LPSpa as a candidate adjuvant for human use and non-clinical evaluation, all *in vivo* and *in vitro* studies, has been ongoing. In the future, we would like to evaluate the efficacy of LPSpa-adjuvanted vaccines in pre-clinical monkey studies followed by the development of safer vaccines for humans.
